# Obesity-Independent Association between Glycemic Status and the Risk of Hematologic Malignancy: A Nationwide Population-Based Longitudinal Cohort Study

**DOI:** 10.3390/cancers13194760

**Published:** 2021-09-23

**Authors:** Jihun Kang, Sang-Man Jin, Seok Jin Kim, Dahye Kim, Kyungdo Han, Su-Min Jeong, JiWon Chang, Sang Youl Rhee, Taewoong Choi, Dong Wook Shin

**Affiliations:** 1 Department of Family Medicine, Kosin University Gospel Hospital, Kosin University College of Medicine, Busan 49267, Korea; josua85@kosinmed.or.kr; 2 Division of Endocrinology and Metabolism, Department of Medicine, Samsung Medical Center, Sungkyunkwan University School of Medicine, Seoul 06351, Korea; 3 Division of Hematology-Oncology, Department of Medicine, Samsung Medical Center, Sungkyunkwan University School of Medicine, Seoul 06351, Korea; kstwoh@skku.edu; 4 Department of Medical Statistics, The Catholic University of Korea, Seoul 03083, Korea; dhkim373@daewoong.co.kr; 5 Department of Statistics and Actuarial Science, Soongsil University, Seoul 06978, Korea; hkd@ssu.ac.kr; 6 Department of Family Medicine, Boramae Medical Center, Seoul Metropolitan Government-Seoul National University, Seoul 07061, Korea; sm2021.jeong@samsung.com; 7 Supportive Care Center, Samsung Medical Center, Seoul 06351, Korea; jiwon.chang@samsung.com; 8 Department of Family Medicine, Samsung Medical Center, Seoul 06351, Korea; 9 Department of Endocrinology and Metabolism, Kyung Hee University School of Medicine, Seoul 02453, Korea; rheesy@khu.ac.kr; 10 Division of Hematologic Malignancies and Cellular Therapy, Duke University Medical Center, Durham, NC 27710, USA; taewoong.choi@duke.edu; 11 Department of Clinical Research Design & Evaluation/Department of Digital Health, Samsung Advanced Institute for Health Science & Technology (SAIHST), Sungkyunkwan University, Seoul 06351, Korea

**Keywords:** hematologic malignancies, Hodgkin’s lymphoma, non-Hodgkin’s lymphoma, diabetes, glycemic status

## Abstract

**Simple Summary:**

The present nationwide population-based longitudinal cohort study showed that diabetes was associated with an increased risk of hematologic malignancies independent of obesity. The risk of NHL increased according to the progression of dysglycemia towards a longer diabetes duration, while HL did not.

**Abstract:**

There have been conflicting results regarding the association between diabetes and the risk of hematologic malignancies, and its interaction with obesity is unknown. This study determined the risk of hematologic malignancies according to the glycemic status in a population-based study involving health screening 9,774,625 participants. The baseline glycemic status of the participants was categorized into no diabetes, impaired fasting glucose (IFG), newly detected diabetes, diabetes duration <5 years, and diabetes duration ≥5 year groups. The risks of overall and specific hematologic malignancies were estimated using a Cox regression analysis. During a median follow up of 7.3 years, 14,733 hematologic malignancies developed. The adjusted hazard ratio (aHR) for the risk of all the hematologic malignancies was 0.99 (95% confidence interval (CI) 0.95–1.02) for IFG, 0.99 (95% CI 0.91–1.08) for newly detected diabetes, 1.03 (95% CI 0.96–1.11) for diabetes duration <5 years, and 1.11 (95% CI 1.03, 1.20) for diabetes duration ≥5 year groups. The association was independent from obesity. The risk of non-Hodgkin’s lymphoma (NHL) increased according to the progression of dysglycemia towards a longer diabetes duration, while Hodgkin’s lymphoma did not. This study in Korea demonstrated diabetes to be associated with an increased risk of hematologic malignancies independent of obesity. The NHL risk increased with the diabetes duration.

## 1. Introduction

Diabetes substantially increases the risk of cardiovascular diseases and mortality. There is also an accumulating body of evidence indicating that diabetes is associated with an increased risk of various solid cancers, including liver [1], endometrial [2], stomach [3], and colorectal cancers [4]. However, there are conflicting results regarding the association between diabetes and hematologic malignancies. Some studies have found that diabetes was associated with an increased risk of HL, while others failed to show an association between these two conditions [5,6,7,8]. There have also been conflicting results regarding non-Hodgkin’s lymphoma (NHL). While some studies showed no association between diabetes and NHL [3,5,6,9,10,11], others [7,12,13] found up to a two-fold higher risk or NHL in diabetes patients (compared to those without diabetes), respectively. Therefore, there has been a lot of heterogeneity in the risk estimates among prior studies. The discrepant results were also observed in myeloid leukemia (ML) and lymphoid leukemia (LL). A positive association between diabetes, ML, and LL was observed in an earlier study [3]. However, others have failed to show a significant association between these conditions [11,14]. The inconsistency of association also existed when the risk for overall leukemia was estimated in previous studies [5,6,7,9,10,12]. Some studies have also found an increased risk of MM among people with diabetes compared to that in patients without diabetes [3,12], while others have not demonstrated this association [5,6,7,9,10,14].

The discrepancies among the previous studies can be explained by several limitations in their study designs. First, the glycemic status at the baseline was dichotomously defined in most studies [3,5,6,7,9,13,14,15,16] rather than discriminating patients based on the broad diabetic spectrum, such as normal glucose tolerance, prediabetes, and diabetes with shorter and longer durations. Therefore, this dichotomous designation prevented prior studies from analyzing the dose-response association between exposure to dysglycemia and the risk of hematologic malignancies. Importantly, the dichotomous definition of diabetes without consideration of disease duration could also result in surveillance bias, because frequent screening and healthcare contacts are likely to happen in the first few years of a diabetes diagnosis. Second, some of the previous studies identified diabetes based on self-reports [6,16]. However, undiagnosed diabetes comprises a substantial proportion of the diabetes population. Third, potential confounders, such as alcohol, smoking, and body mass index, were not adjusted for in the majority of the previous studies [3,7,9,12,15]. Furthermore, to our knowledge, the vast majority of the previous studies included limited numbers of nonobese people with diabetes, because there has been only limited data in the Asian population, except the few studies conducted in China [9,13,15].

The present nationwide population-based longitudinal cohort study determined the risk of overall and specific hematologic malignancies according to the baseline glucose tolerance status and diabetes disease duration after adjusting for potential confounders, including the BMI. To avoid surveillance bias, the study was conducted in those who participated in the universal national health screening program, which has been designed to represent the entire general population in Korea, where the proportion of nonobese type 2 diabetes among the diabetes population is relatively high.

## 2. Materials and Methods

### 2.1. Data Source and Study Participants

**Figure 1:**
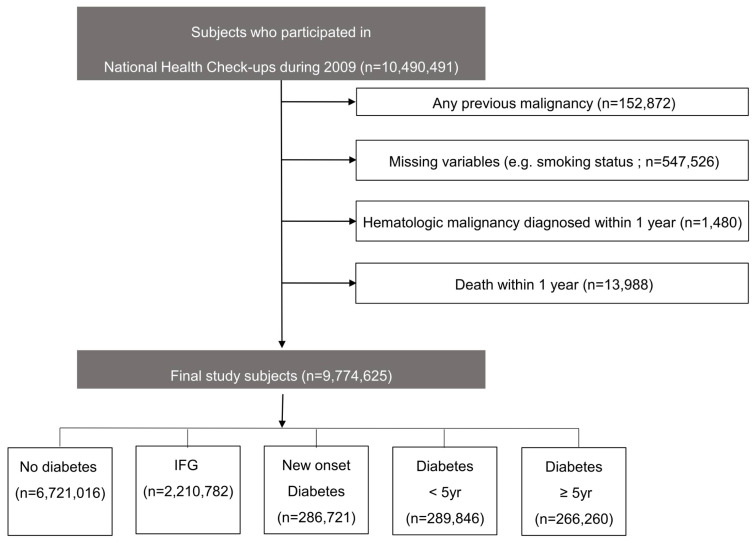
Flowchart of the study protocol and participants. IFG, impaired fasting glucose.

The present study used the Korean National Health Insurance Services (KNHIS) database. The KNHIS is a universal healthcare program that covers 97% of the entire Korean population. The remaining 3% is covered by the Medicaid program. The KNHIS biennially provides a national health screening program for all Korean employees aged ≥20 and individuals aged ≥40, regardless of their employment status. This nationwide health examination comprises anthropometric indices; biochemical tests; and a questionnaire regarding health behaviors (such as alcohol consumption, smoking status, and physical activity). We identified 10,490,491 individuals who participated in this screening program in 2009. Among them, we excluded 715,866 participants for the following reasons: previous cancer diagnosis (*N* = 152,872); missing data (*N* = 547,526); development of a hematologic malignancy (*N* = 1480), or death (*N* = 13,988) within 1 year of enrollment. Therefore, a total of 9,774,625 participants were ultimately included in the analysis (Figure 1). The study protocol was approved by the Institutional Review Board of Samsung Medical Center (SMC 2018-08-112). Informed consent was waived, because anonymized and deidentified data were used.

### 2.2. Definition of Diabetes and Glucose Tolerance Status

The definition of diabetes was defined by one or more of the following: the presence of the diagnosis codes E11.x–E14.x from the 10th revision of the International Statistical Classification of Diseases (ICD-10), prescription(s) of oral and/or injectable antidiabetic medications, or a fasting plasma glucose ≥ 126 mg/dL [17] during the national health screening program. This definition of diabetes has been described elsewhere [18]. Impaired fasting glucose (IFG) was defined as a fasting glucose level of 100–125 mg/dL [17]. Based on their glycemic status, the participants were categorized into five groups, as follows: no diabetes (fasting plasma glucose <100 mg/dL), IFG (fasting glucose 100–125 mg/dL), newly detected diabetes, diabetes duration <5 year, and diabetes duration ≥5 year [18]. Their glycemic status during the 2009 national health screening was defined as the baseline glycemic status.

### 2.3. Definition of Hematologic Malignancies

The primary outcome was incident hematologic cancer. The incidence of hematologic malignancies was identified using the registered diagnosis code from the 10th revision of the International Statistical Classification of Diseases (ICD-10), as defined in our previous study [19]: HL (C810; NHL (Diffuse Large B-cell lymphoma (C83.3) and follicular lymphoma (C82)); ML (chronic myeloid leukemia (C92.1) and acute myeloid leukemia (C92.0, C92.5, C92.4, C92.6, C93.0, C94.0, and C94.2)); LL (chronic lymphocytic leukemia (C91.1) and acute lymphocytic leukemia (C91.0)); and MM (C90.0). We also matched the code-based diagnosis with registration to the critical disease copayment reduction program to ensure the accuracy of the cancer diagnosis (since patients can only be registered in this program if they have been diagnosed with cancer by a physician). We followed the participants from the day of health screening in 2009 to the date of a hematologic malignancy diagnosis, death from any cause, or 31 December 2017, whichever came first.

### 2.4. Covariates

The smoking status was categorized into the following three groups: current, ex-, and nonsmokers. Alcohol consumption was categorized into none, mild (1–29 g/day), and heavy intake (≥30 g/day). Regular physical activity was defined as strenuous exercise ≥1 session/week for at least 20 minutes per occasion. The body mass index (BMI) was calculated by kg/m^2^. Hypertension and dyslipidemia were defined by the anthropometric results (blood pressure ≥ 140 mmHg or total cholesterol ≥240 mg/dL) or ICD-10 codes (I10-I13 and I15 or E78) associated with the relevant medications (antihypertensive or lipid-lowering agents).

### 2.5. Statistical Analysis

The baseline characteristics of the study participants (among the five groups) were compared using the chi-square test for categorical variables and the analysis of variance (ANOVA) for continuous variables. We calculated the incidence rate of the hematologic malignancies as the total incident cases divided by 100,000 person-years (P-y). The risk of hematologic malignancies was evaluated using a Cox regression analysis. The covariates were adjusted as follows: Model 1 was adjusted for age and sex; Model 2 was additionally adjusted for the smoking status, alcohol consumption, physical activity, and BMI. A stratified analysis was conducted to explore the effect modification by age and BMI. Separate regressions for each age and BMI group were performed. We also tested the *p* for interactions by inserting the interaction term (age/BMI × glycemic status) with the age/BMI and the glycemic status in the analysis models. Several sensitivity analyses were conducted: (1) including patients who developed hematologic malignancy (*N* = 1480) or death (*N* = 13,988) within 1 year of enrollment to reduce the chance of reverse causality, (2) limiting the patients to a subgroup of newly diagnosed diabetes patients to test whether the risk of hematologic malignancies was rapidly elevated in the first year of a diabetes diagnosis, and (3) additionally adjusting diabetes medications to estimate the effect of these medications on the risk of hematologic malignancies among diabetes patients. The statistical analyses were conducted using SAS version 9.4 (SAS Institute Inc., Cary, NC, USA). Two-tailed *p*-values < 0.05 were considered statistically significant.

## 3. Results

The baseline characteristics of the study participants are described in Table 1. During the median follow-up of 7.3 years (interquartile range 7.1–7.6 year), 14,733 hematologic cancers were developed. Among these malignancies, there were 694 cases of HL, 5492 cases of NHL, 4269 cases of ML, 1212 cases of LL, and 3615 cases of MM.

### 3.1. Risk of Hematologic Malignancies According to the Presence of Diabetes

The incidence rates for all the hematologic malignancies with and without diabetes were 35.2 and 19.4 per 100,000 person-year, respectively, with an adjusted hazard ratio (aHR) 1.05 (95% CI (1.01–1.10). The presence of diabetes was associated with a higher risk for HL (aHR 1.28, 95% CI 1.03–1.59) and NHL (aHR 1.09, 95% CI 1.01–1.17) compared to that in the no diabetes group. There was no significant difference in ML (aHR 1.06, 95% CI 0.97–1.16), LL (aHR 1.01, 95% CI 0.85–1.20), or MM (aHR 0.99, 95% CI 0.90–1.08) risks between the two groups (Table 2).

### 3.2. Risk of Hematologic Malignancies According to Baseline Glucose Tolerance Status and Diabetes Duration

The incidence rates of all hematologic malignancies were 18.1, 23.4, 26.5, 35.7, and 43.9 for the no diabetes, IFG, newly detected diabetes, diabetes < 5 year, and diabetes ≥ 5 year groups, with aHRs (95% CI) of 0.99 (0.95–1.02), 0.99 (0.91–1.08), 1.03 (0.96–1.11), and 1.11 (1.03–1.20), respectively.

**Figure 2:**
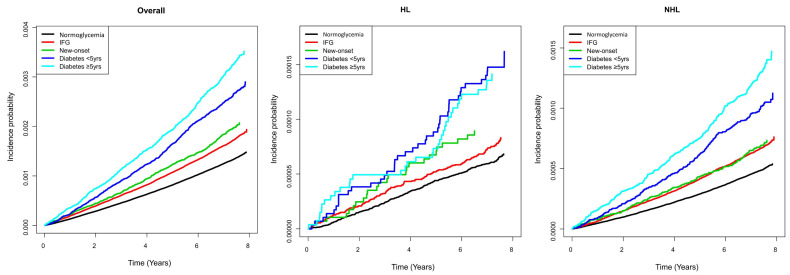
Cumulative incidence of the overall hematologic malignancy, Hodgkin’s lymphoma, and non-Hodgkin’s lymphoma, according to the baseline glycemic status. IFG, impaired fasting glucose; HL, Hodgkin’s lymphoma; NHL, non-Hodgkin’s lymphoma.

The risk of HL was significantly higher in the diabetes < 5 year group (aHR 1.51, 95% CI 1.09–2.09) than it was in the no diabetes group but not in the diabetes ≥ 5 year group (aHR 1.27, 95% CI 0.89–1.81). The risk of NHL was significantly higher in the diabetes ≥ 5 year group (aHR 1.24, 95% CI 1.10–1.39) than it was in the no diabetes group. The cumulative incidences of HL and NHL according to the glucose tolerance status is shown in Figure 2. There were no significant differences in the risk of MM, LL, and ML based on the baseline glycemic status (Table 3).

### 3.3. Glycemic Status and Risk of Hematologic Malignancies Stratified by Age and BMI

There was a significant interaction with age, but not with BMI, in the association between the glycemic status and the risk of all hematologic malignancies (Table 4). The significant interaction with age in the association between the glycemic status and the risk of all hematologic malignancies was attributable to the significant association between the diabetes <5 year (aHR 1.23, 95% CI 1.04–1.44) and diabetes ≥ 5 year groups (aHR 1.26, 95% CI 1.06–1.50) and the risk of NHL in the participants with ages 40–64 years and a small but significant association between the diabetes ≥ 5 year group and the risk of ML in the participants with ages < 40 years (aHR 6.06, 95% CI 2.5–68).

### 3.4. Sensitivity Analyses

We conducted an analysis including individuals diagnosed with hematologic malignancies within 1 year (*n* = 1480) and death within 1 year (*n* = 13,988), and the results were similar to those of our main analysis (Tables S1 and S2). Furthermore, the sensitivity analysis including only newly diagnosed diabetes in the 2009 screening showed no abrupt increase in incidence of the overall hematologic malignancy, HL, or NHL during the first year of a diabetes diagnosis (Figure S1). Finally, subgroup analyses with diabetes patients showed that further adjustments of diabetes medications did not change the associations (Table S3).

## 4. Discussion

To our knowledge, this is the first study that investigated the risk of a wide range of hematologic malignancies according to the broad diabetic spectrum, such as normal glucose tolerance, prediabetes, and diabetes with shorter and longer durations after a robust adjustment for potential confounders, including the BMI. This large-scale nationwide population-based cohort study revealed an increasing trend of the risk of all hematologic malignancies and NHL according to the progression of the glycemic status toward diabetes with a longer disease duration, without a significant interaction with obesity.

The present study suggested that the risk of NHL was linearly and positively associated with the progression of the glycemic status toward diabetes with a longer disease duration. This finding was consistent with a previous study [20], in which participants with diabetes for >3.5 years had a higher risk for NHL (aHR 1.17 95% CI 1.03–1.33) than those with diabetes ≤ 3.5 years did (who had no increased risk of NHL, aHR 0.94 95% CI 0.82–1.08) [9]. An Israelite study showed that the highest risk of NHL was in the first year of a diabetes diagnosis (aHR 5.53 95% CI 5.01–6.10) and no significant association in the following year (aHR 1.24 95% CI 0.98–1.58 in 1 to 2 years after the diagnosis) and, again, an increased risk in the subsequent years (aHR 1.41 95% CI 1.25–1.59 in 2–11 years) [12]. The highest risk of NHL in the first year of a diabetes diagnosis in the study of Israelite might be due to a further diagnostic work-up and frequent healthcare contacts immediately after the diagnosis of diabetes. However, our analysis avoided surveillance bias, because we compared the risk of NHL using the baseline glycemic status of each participant of the national health screening program. In addition, our sensitivity analyses without a 1-year lag period did not find a higher risk in the first year of a diabetes diagnosis.

The current study showed an increased incidence of HL among people with diabetes, when compared to those without diabetes. However, it turned out to be attributable to the increased incidence of HL among people with diabetes with a shorter duration, failing to show an increasing trend of the risk of HL according to the progression of glycemic status toward diabetes with a longer disease duration. An earlier meta-analysis reported no association between diabetes and HL [8]. Other studies have also failed to show a significant association between diabetes and incident HL [5,6,7]. Given the low incidence of HL, which is around one-tenth of NHL [21], and that the majority of HL occurs before age 40, the age distribution of a study population is very important to detect a significant difference in the risk of HL in relation to diabetes. However, most previous studies, including one large-scale Australian study, only included participants aged > 40 years [3,6,14]. Therefore, it is difficult to find evident differences regarding the HL risk between patients with and without diabetes. In contrast, our study included a large number of participants with <40 (*N* = 3,099,268) years of age and provided an improved power to test the relationship between diabetes and HL. In addition, unlike in Western countries where there is a bimodal incidence pattern of HL, the peak incidence of HL in Korea is 19–30 years [22]. We speculate that the overall increase in the incidence of HL in those with diabetes in the present study should be confirmed by further studies and meta-analyses, including a sufficient number of participants <40 years of age, with consideration of the baseline glucose tolerance status and diabetes duration.

Our study found no significant association between diabetes and the risk of other specific hematologic malignancies, such as ML, LL, and MM. Although there was a small but significant association between the diabetes ≥ 5 year group and the risk of ML in the participants with ages < 40 years, it was difficult to draw a conclusion because of the small event number. Two studies from in Italy and England also both reported that diabetes was not associated with an elevated risk of LL [11,14]. In addition, although the risk of ML and LL were not presented separately, the overall risk of leukemia among the individuals with diabetes was not different from that of the general population in Taiwan [9]. A meta-analysis showed no significant association between diabetes and MM (OR 1.22, 95% CI 0.98–1.53) [8], which was consistent with our findings.

Several possible mechanisms have been proposed regarding the association between diabetes and hematologic malignancies. First, chronic exposure to hyperinsulinemia plays an important role in tumor development and progression by modulating insulin-like growth factor-1 (IGF-1) activity. Insulin inhibits the production of IGF-1-binding proteins from the liver [23], which leads to an increased concentration of biologically active free IGF-1, which is involved in tumor promotion. Second, increased insulin levels can trigger the IGF-1 signaling pathway that subsequently activates phosphoinositide 3 kinase (PI3K) and mitogen-activated protein kinase (MAPK). These two kinases ultimately influence the cell survival and tumor development [24,25]. This hypothesis was partly supported by a recent prospective epidemiologic study, which found a positive tendency between plasma IGF-1 and diffuse larger B-cell lymphoma, although a statistical significance was not reached [26]. Second, the microangiopathy of the bone marrow niche in diabetes could be attributable to the risk of hematologic malignancies. An experimental study of mice demonstrated that the bone marrow of mice with diabetes showed a more fragmented microvascular structure and reduced cellularity and perfusion compared with the bone marrow of the control [27]. Third, diabetes is accompanied frequently by hyperleptinemia as a result of leptin resistance, and leptin signaling could participate in the pathogenesis of hematologic malignancy. A complex interplay among obesity, fat mass, adipokines, and the risk of hematologic malignancy has been suggested [28,29]. In addition, oxidative stress, which is associated with chronic inflammation, might promote the development of hematologic malignancies. Several biomarkers such as malondialdehyde, superoxide anion, and H_2_O_2_ were elevated in hematologic malignancies. These biomarkers imply that oxidative stress contributes to the development of these malignancies [30].

Notably, the BMI did not affect the association between diabetes and the risk of hematologic malignancies in our study. Since people with diabetes in East Asia have a lower mean BMI than those in more Western countries [31], we had the statistical power to determine if the risk of hematologic malignancy is equally increased in lean people with diabetes than it is in patients with higher BMIs. Our study demonstrated that the risks of HL and NHL were consistently increased even in people with diabetes with normal BMIs. This finding implies that the pathophysiologic link between diabetes and hematologic malignancy may not be confined to insulin resistance. For example, oxidative stress related to hyperglycemia and glycemic variability could at least, in part, play a contributory role in the development of NHL [30].

This study had several limitations that should be noted. First, we had limited information on the dietary patterns [32] and viral infections, such as human T-cell lymphotropic virus 1, Epstein–Barr virus, and human herpes-virus 8 infection [33,34]. Second, we did not consider the potential role of anti-diabetes medications in the development of hematologic malignancies, because the main focus of this study was to investigate the diabetes duration and hematologic malignances. Third, because the younger age group (20–40 years) was only composed of employed individuals, the issue of under-representativeness could be raised in this age group. Differences in sociodemographic and health profiles between unemployed and employed groups might have an influence on the observed association in the younger group. Fourth, 5.2% of the eligible participants (*N* = 547,526) were excluded from the analyses due to missing information (mainly on smoking, alcohol consumption, and physical activity). The participants with missing information tended to be younger (45.5 vs. 47.2 years) and were more likely to be male (63.2% vs. 54.8%). However, after adjusting for age and sex, there was no difference in the risk of hematologic malignancies between participants with and without missing variables (HR1.06, 95% CI: 0.99–1.13). Furthermore, most of the missing information was alcohol consumption, smoking status, and physical activity; hence, it is unlikely that there was a significant bias in the present study. Fifth, the risk of the NHL subtypes was not estimated due to a lack of detailed information on the pathologic findings. Sixth, our results may not be generalizable to other populations, considering that hematological cancers have different epidemiological patterns and risk profiles across geographic regions and ethnicities [33].

## 5. Conclusions

In conclusion, this large population-based nationally representative study in Korea demonstrated diabetes to be associated with an increased risk of overall hematologic malignancies independent of obesity. The risk of NHL increased according to the progression of dysglycemia toward diabetes with a longer disease duration, while HL did not.

## Figures and Tables

**Table 1:** Baseline characteristics of the study participants.

Glycemic Status Category	Normoglycemia	IFG	Newly Detected Diabetes	Diabetes < 5 Years	Diabetes ≥ 5 Years
	(*n* = 6,721,016)	(*n* = 2,210,782)	(*n* = 286,721)	(*n* = 289,846)	(*n* = 266,260)
Age	44.82 ± 13.84	49.63 ± 13.27	51.85 ± 12.66	58.31 ± 11.09	61.89 ± 9.89
Sex, male	3,471,338 (51.7)	1,370,671 (62.0)	204,232 (71.2)	168,676 (58.2)	145,231 (54.5)
Smoking status					
Non-smoker	4,131,803 (61.5)	1,205,511 (54.5)	133,656 (46.6)	167,006 (57.6)	167,597 (62.9)
Ex-smoker	852,248 (12.7)	385,137 (17.4)	52,303 (18.2)	53,932 (18.6)	47,252 (17.8)
Current-smoker	1,736,965 (25.8)	620,134 (28.1)	100,762 (35.1)	68,908 (23.8)	51,411 (19.3)
Alcohol consumption					
None	3,483,838 (51.8)	1,041,566 (47.1)	123,167 (43.0)	175,294 (60.5)	178,196 (66.9)
Mild	2,772,531 (41.3)	935,802 (42.33)	122,823 (42.8)	88,405 (30.5)	69,817 (26.2)
Heavy	464,647 (6.9)	233,414 (10.6)	40,731 (14.2)	26,147 (9.0)	18,247 (6.9)
Regular physical activity	1,161,028 (17.3)	419,581 (19.0)	54,394 (19.0)	64,325 (22.2)	64,782 (24.3)
Comorbidities					
Hypertension	1,289,796 (19.2)	726,690 (32.9)	124,915 (43.6)	180,358 (62.2)	177,685 (66.7)
Dyslipidemia	938,118 (14.0)	495,844 (22.4)	81,959 (28.6)	140,205 (48.4)	125,602 (47.2)
Chronic kidney disease	326,778 (4.86)	147,030 (6.65)	21,675 (7.56)	31,530 (10.88)	45,848 (17.22)
Obesity (BMI > 25 kg/m^2^)	1,860,854 (27.7)	875,575 (39.6)	137,675 (48.0)	150,114 (51.8)	114,271 (42.9)
Body mass index, kg/m^2^	23.1 (21.1–25.3)	24.2 (22.2–26.3)	24.9 (22.8–27.0)	25.2 (23.2–27.3)	24.5 (22.6–26.5)
Waist circumference, cm	79 (72–85)	82 (76–88)	85 (80–90)	86 (81–91)	85 (80–90)
Fasting glucose, mg/dL	88 (82–94)	106 (102–112)	140 (131–159)	126 (107–153)	135 (112–169)
Systolic blood pressure, mmHg	120 (110–130)	125 (116–135)	130 (120–139)	130 (120–138)	130 (120–139)
Diastolic blood pressure, mmHg	75 (70–80)	80 (70–84)	80 (73–88)	80 (70–85)	80 (70–83)
Total cholesterol, mg/dL	190 (168–214)	199 (176–225)	205 (179–233)	192 (166–221)	185 (160–213)
Triglycerides, mg/dL	105.4 (105.4–105.4)	128.6 (128.5–128.7)	162.1 (161.8–162.5)	150.5 (150.2–150.8)	139.7 (139.4–140.0)
HDL-cholesterol, mg/dL	54 (46–64)	53 (45–62)	51 (43–60)	49 (41–58)	49 (41–57)
LDL-cholesterol, mg/dL	110 (90–132)	116 (94–139)	115 (91–141)	108 (83–133)	104 (81–128)
Sulfonylurea				203,200 (70.1)	224,755 (84.4)
Metformin				200,405 (69.1)	193,325 (72.6)
Meglitinides				10,024 (3.5)	14,888 (5.6)
Thiazolidinedione				31,763 (11.0)	40,912 (15.4)
Dipeptidyl peptidase-4 inhibitor				26,015 (9.0)	23,424 (8.8)
Alpha-glucosidase inhibitor				39,788 (13.7)	79,722 (29.9)
Insulin				23,523 (8.1)	47,469 (17.8)

Data were presented as the numbers (%) or means (95% confidence interval). IFG, impaired fasting glucose; BMI, body mass index; HDL, high density lipoprotein; LDL, low density lipoprotein.

**Table 2:** The risk of hematologic malignancies among the participants with diabetes.

	Subjects (*N*)	Event (*n*)	Duration (Person-Years)	Incidence Rate (per 100,000 Person-Years)	Model 1	Model 2
All Hematologic Malignancy						
No Diabetes	8,931,798	12,632	65,014,611.6	19.40	1 (Ref.)	1 (Ref.)
Diabetes	842,827	2101	5,974,211.7	35.20	1.08 (1.03,1.13)	1.05 (1.01,1.10)
1. Hodgkin Lymphoma						
No Diabetes	8,931,798	592	65,041,866.8	0.91	1 (Ref.)	1 (Ref.)
Diabetes	842,827	102	5,978,296.2	1.71	1.27 (1.02,1.58)	1.28 (1.03,1.59)
2. Non-Hodgkin Lymphoma						
No Diabetes	8,931,798	4691	65,031,545.4	7.21	1 (Ref.)	1 (Ref.)
Diabetes	842,827	801	5,976,673.7	13.40	1.11 (1.03,1.20)	1.09 (1.01,1.17)
3. Myeloid Leukemia						
No Diabetes	8,931,798	3698	65,035,921.5	5.69	1 (Ref.)	1 (Ref.)
Diabetes	842,827	571	5,977,584.1	9.55	1.09 (1.00,1.19)	1.06 (0.97,1.16)
4. Lymphoid Leukemia						
No Diabetes	8,931,798	1059	65,041,338.5	1.63	1 (Ref.)	1 (Ref.)
Diabetes	842,827	153	5,978,245.5	2.56	1.04 (0.87,1.23)	1.01 (0.85,1.20)
5. Multiple Myeloma						
No Diabetes	8,931,798	3,067	65,037,282.2	4.72	1 (Ref.)	1 (Ref.)
Diabetes	842,827	548	5,977,526.9	9.17	1.01 (0.92,1.10)	0.99 (0.90,1.08)

Model 1 was adjusted for age and sex. Model 2 was additionally adjusted for age, sex, smoking, drinking, physical activity, and body mass index.

**Table 3:** The risk of hematologic malignancies according to the glycemic status and diabetes duration.

	Subjects (*N*)	Event (*n*)	Duration (Person-Years)	Incidence Rate (per 1000 Person-Years)	Model 1	Model 2
All Hematologic Malignancy						
Normoglycemia	6,721,016	8885	48,998,241.0	18.13	1 (Ref.)	1 (Ref.)
Impaired fasting glucose	2,210,782	3747	16,016,370.5	23.40	1.00 (0.96,1.04)	0.99 (0.95,1.02)
Newly detected diabetes	286,721	543	2,046,567.9	26.53	1.01 (0.92,1.10)	0.99 (0.91,1.08)
Diabetes (<5 years)	289,846	740	2,067,385.4	35.79	1.07 (0.99,1.15)	1.03 (0.96,1.11)
Diabetes (≥5 years)	266,260	818	1,860,258.4	43.97	1.14 (1.06,1.23)	1.11 (1.03,1.20)
1. Hodgkin’s Lymphoma						
Normoglycemia	6,721,016	422	49,017,433.3	0.86	1 (Ref.)	1 (Ref.)
Impaired fasting glucose	2,210,782	170	16,024,433.5	1.06	1 (0.84,1.20)	1.02 (0.85,1.22)
Newly detected diabetes	286,721	25	2,047,711.7	1.22	1.03 (0.69,1.54)	1.04 (0.69,1.57)
Diabetes (<5 years)	289,846	42	2,068,831.9	2.03	1.49 (1.08,2.06)	1.51 (1.09,2.09)
Diabetes (≥5 years)	266,260	35	1,861,752.6	1.88	1.26 (0.89,1.79)	1.27 (0.89,1.81)
2. Non-Hodgkin’s Lymphoma						
Normoglycemia	6,721,016	3239	49,010,295.7	6.61	1 (Ref.)	1 (Ref.)
Impaired fasting glucose	2,210,782	1452	16,021,249.7	9.06	1.07 (1.00,1.14)	1.05 (0.98,1.12)
Newly detected diabetes	286,721	191	2,047,292.1	9.33	0.98 (0.84,1.13)	0.95 (0.82,1.11)
Diabetes (<5 years)	289,846	282	2,068,219.3	13.64	1.13 (1.00,1.28)	1.09 (0.96,1.23)
Diabetes (≥5 years)	266,260	328	1,861,162.3	17.62	1.27 (1.13,1.43)	1.24 (1.10,1.39)
3. Myeloid Leukemia						
Normoglycemia	6,721,016	2667	49,013,083.3	5.44	1 (Ref.)	1 (Ref.)
Impaired fasting glucose	2,210,782	1031	16,022,838.2	6.44	0.95 (0.88,1.02)	0.93 (0.86,1.00)
Newly detected diabetes	286,721	150	2,047,485.0	7.33	0.97 (0.82,1.15)	0.94 (0.80,1.11)
Diabetes (<5 years)	289,846	197	2,068,637.3	9.52	1.05 (0.91,1.21)	1.00 (0.86,1.16)
Diabetes (≥5 years)	266,260	224	1,861,461.8	12.03	1.18 (1.03,1.36)	1.14 (0.99,1.31)
4. Lymphoid leukemia						
Normoglycemia	6,721,016	746	49,017,148.6	1.52	1 (Ref.)	1 (Ref.)
Impaired fasting glucose	2,210,782	313	16,024,189.8	1.95	1.05 (0.92,1.19)	1.02 (0.90,1.17)
Newly detected diabetes	286,721	45	2,047,662.3	2.20	1.07 (0.79,1.44)	1.04 (0.77,1.40)
Diabetes (<5 years)	289,846	54	2,068,839.1	2.61	1.05 (0.79,1.39)	1.01 (0.76,1.33)
Diabetes (≥5 years)	266,260	54	1,861,744.1	2.90	1.04 (0.79,1.38)	1.02 (0.77,1.35)
5. Multiple Myeloma						
Normoglycemia	6,721,016	2142	49,014,251.6	4.37	1 (Ref.)	1 (Ref.)
Impaired fasting glucose	2,210,782	925	16,023,030.6	5.77	0.96 (0.89,1.04)	0.96 (0.89,1.04)
Newly detected diabetes	286,721	144	2,047,492.8	7.03	1.03 (0.87,1.21)	1.02 (0.86,1.21)
Diabetes (<5 years)	289,846	199	2,068,565.9	9.62	1.01 (0.87,1.17)	0.98 (0.85,1.14)
Diabetes (≥5 years)	266,260	205	1,861,468.3	11.01	0.96 (0.83,1.11)	0.93 (0.81,1.08)

Model 1 was adjusted for age and sex. Model 2 was additionally adjusted for age, sex, smoking, drinking, physical activity, and body mass index.

**Table 4:** Diabetes and glycemic status and risk of hematologic malignancies stratified by age and the body mass index (BMI).

		*N*	All Blood Cancer		Hodgkin Lymphoma		Non-Hodgkin Lymphoma		Myeloid Leukemia		Lymphoid Leukemia		Multiple Myeloma	
			Event	aHR (95% CI)	Event	aHR (95% CI)	Event	aHR (95% CI)	Event	aHR (95% CI)	Event	aHR (95% CI)	Event	aHR (95% CI)
Age	Diabetes													
<40	No	3,035,864	1446	1 (Ref.)	132	1 (Ref.)	521	1 (Ref.)	604	1 (Ref.)	162	1 (Ref.)	76	1 (Ref.)
	Yes	63,404	45	1.26 (0.94,1.70)	3	1.15 (0.36,3.65)	12	0.93 (0.52,1.65)	24	1.56 (1.03,2.35)	4	1.06 (0.39,2.88)	3	1.45 (0.45,4.65)
40–64	No	4,895,306	7197	1 (Ref.)	313	1 (Ref.)	2711	1 (Ref.)	2109	1 (Ref.)	633	1 (Ref.)	1729	1 (Ref.)
	Yes	528,531	1104	1.10 (1.03,1.17)	56	1.25 (0.93,1.67)	430	1.15 (1.04,1.28)	308	1.09 (0.96,1.23)	86	1.02 (0.81,1.28)	266	1.01 (0.89,1.16)
≥65	No	1,000,628	3989	1 (Ref.)	147	1 (Ref.)	1459	1 (Ref.)	985	1 (Ref.)	264	1 (Ref.)	1262	1 (Ref.)
	Yes	250,892	952	0.96 (0.89,1.03)	43	1.22 (0.86,1.71)	359	0.98 (0.87,1.10)	239	0.97 (0.84,1.12)	63	0.97 (0.73,1.28)	279	0.90 (0.79,1.03)
*p* for interaction				0.004		0.706		0.078		0.093		0.896		0.154
	Glycemic status													
<40	Normoglycemia	2,528,330	1179	1 (Ref.)	105	1 (Ref.)	426	1 (Ref.)	482	1 (Ref.)	141	1 (Ref.)	64	1 (Ref.)
	Impaired fasting glucose	507,534	267	1.02 (0.89,1.17)	27	1.35 (0.88,2.08)	95	1.01 (0.80,1.26)	122	1.12 (0.92,1.37)	21	0.69 (0.43,1.09)	12	0.78 (0.42,1.46)
	Newly detected diabetes	47,588	31	1.19 (0.83,1.70)	2	1.10 (0.27,4.50)	10	1.05 (0.56,1.98)	14	1.28 (0.75,2.18)	3	0.99 (0.31,3.12)	3	1.87 (0.58,6.03)
	Diabetes < 5 y	12,588	7	0.94 (0.44,1.97)	1	2.06 (0.28,14.90)	1	0.37 (0.05,2.61)	5	1.55 (0.64,3.77)	0	N/A	0	N/A
	Diabetes ≥ 5 y	3228	7	3.64 (1.73,7.66)	0	N/A	1	1.41 (0.20,10.08)	5	6.06 (2.50,14.68)	1	4.73 (0.66,34.09)	0	N/A
40–64	Normoglycemia	3,519,512	4999	1 (Ref.)	221	1 (Ref.)	1848	1 (Ref.)	1507	1 (Ref.)	430	1 (Ref.)	1203	1 (Ref.)
	Impaired fasting glucose	1,375,794	2198	1.00 (0.95,1.05)	92	0.92 (0.721.18)	863	1.06 (0.98,1.16)	602	0.91 (0.83,1.00)	203	1.09 (0.92,1.29)	526	0.98 (0.88,1.08)
	Newly detected diabetes	190,421	342	1.05 (0.94,1.17)	15	0.95 (0.56,1.62)	125	1.05 (0.87,1.25)	91	0.93 (0.75,1.16)	28	1.02 (0.69,1.50)	90	1.13 (0.91,1.41)
	Diabetes < 5 y	187,609	409	1.12 (1.01,1.24)	22	1.33 (0.85,2.08)	162	1.23 (1.04,1.44)	114	1.07 (0.88,1.30)	33	1.11 (0.77,1.59)	100	1.03 (0.84,1.27)
	Diabetes ≥ 5 y	150,501	353	1.12 (1.00,1.25)	19	1.36 (0.85,2.19)	143	1.26 (1.06,1.50)	103	1.16 (0.95,1.43)	25	1.01 (0.67,1.52)	76	0.86 (0.68,1.09)
≥65	Normoglycemia	673,174	2707	1 (Ref.)	96	1 (Ref.)	965	1 (Ref.)	678	1 (Ref.)	175	1 (Ref.)	875	1 (Ref.)
	Impaired fasting glucose	327,454	1282	0.94 (0.88,1.01)	51	1.07 (0.76,1.51)	494	1.01 (0.90,1.12)	307	0.89 (0.78,1.02)	89	1.01 (0.78,1.31)	387	0.89 (0.79,1.01)
	Newly detected diabetes	48,712	170	0.84 (0.72,0.98)	8	1.13 (0.55,2.33)	56	0.77 (0.58,1.00)	45	0.87 (0.64,1.18)	14	1.08 (0.62,1.86)	51	0.80 (0.60,1.06)
	Diabetes < 5 y	89,649	324	0.89 (0.79,1.00)	19	1.55 (0.94,2.55)	119	0.90 (0.74,1.09)	78	0.85 (0.67,1.07)	21	0.90 (0.57,1.42)	99	0.86 (0.69,1.06)
	Diabetes ≥ 5 y	112,531	458	1.03 (0.93,1.14)	16	1.06 (0.62,1.80)	184	1.15 (0.98,1.34)	116	1.03 (0.85,1.26)	28	0.98 (0.66,1.47)	129	0.91 (0.76,1.09)
*p* for interaction				0.001		0.977		0.227		0.008		0.51		0.368
BMI, kg/m^2^	Diabetes													
<25	No	6,195,369	8174	1 (Ref.)	396	1 (Ref.)	2995	1 (Ref.)	2380	1 (Ref.)	680	1 (Ref.)	2025	1 (Ref.)
	Yes	440,767	1092	1.07 (1.00,1.14)	55	1.29 (0.97,1.72)	409	1.11 (1.00,1.23)	282	1.03 (0.91,1.17)	89	1.18 (0.94,1.48)	294	1.00 (0.88,1.13)
≥25	No	2,736,429	4458	1 (Ref.)	196	1 (Ref.)	1696	1 (Ref.)	1318	1 (Ref.)	379	1 (Ref.)	1042	1 (Ref.)
	Yes	402,060	1009	1.05 (0.98,1.13)	47	1.22 (0.88,1.70)	392	1.07 (0.96,1.20)	289	1.12 (0.99,1.28)	64	0.86 (0.65,1.12)	254	1.00 (0.87,1.15)
*p* for interaction				0.849		0.957		0.875		0.544		0.073		0.879
	Glycemic status													
<25	Normoglycemia	4,860,162	5990	1 (Ref.)	288	1 (Ref.)	2148	1 (Ref.)	1789	1 (Ref.)	504	1 (Ref.)	1477	1 (Ref.)
	Impaired fasting glucose	1,335,207	2184	1.00 (0.95,1.05)	108	1.09 (0.87,1.37)	847	1.09 (1.00,1.18)	591	0.94 (0.85,1.03)	176	1.01 (0.85,1.20)	548	0.95 (0.86,1.05)
	Newly detected diabetes	149,046	275	0.97 (0.86,1.10)	13	1.03 (0.59,1.80)	86	0.86 (0.69,1.07)	72	0.90 (0.71,1.13)	33	1.51 (1.06,2.15)	77	1.05 (0.80,1.26)
	Diabetes < 5 y	139,732	351	1.044 (0.94,1.16)	22	1.64 (1.05,2.54)	137	1.16 (0.97,1.38)	83	0.91 (0.73,1.14)	23	0.93 (0.61,1.42)	100	1.01 (0.82,1.23)
	Diabetes ≥ 5 y	151,989	466	1.16 (1.05,1.28)	20	1.31 (0.82,2.07)	186	1.32 (1.13,1.54)	127	1.19 (0.99,1.43)	33	1.14 (0.80,1.63)	117	0.95 (0.78,1.15)
≥25	Normoglycemia	1,860,854	2895	1 (Ref.)	134	1 (Ref.)	1091	1 (Ref.)	878	1 (Ref.)	242	1 (Ref.)	665	1 (Ref.)
	Impaired fasting glucose	875,575	1563	0.98 (0.92,1.04)	62	0.88 (0.65,1.19)	605	1.00 (0.91,1.11)	440	0.94 (0.84,1.06)	137	1.06 (0.86,1.31)	377	1.00 (0.88,1.13)
	Newly detected diabetes	137,675	268	1.02 (0.90,1.16)	12	1.01 (0.56,1.83)	105	1.05 (0.86,1.28)	78	1.03 (0.82,1.30)	12	0.57 (0.32,1.02)	67	1.07 (0.831.38)
	Diabetes < 5 y	150,114	389	1.04 (0.94,1.160)	20	1.30 (0.81,2.09)	145	1.02 (0.86,1.22)	114	1.12 (0.92,1.37)	31	1.10 (0.76,1.61)	99	0.99 (0.80,1.23)
	Diabetes ≥ 5 y	114,271	352	1.07 (0.96,1.20)	15	1.17 (0.68,2.01)	142	1.15 (0.96,1.37)	97	1.13 (0.91,1.40)	21	0.88 (0.56,1.39)	88	0.94 (0.75,1.18)
*p* for interaction				0.862		0.834		0.302		0.673		0.045		0.973

aHR; adjusted hazard ratio; confidence interval. The analysis models were adjusted for age, sex, smoking, drinking, physical activity, and body mass index.

## Data Availability

The data that support the findings of this study are available from the National Health Insurance Corporation, but restrictions apply to the availability of these data, which were used under license NHIS-2019-1-130 for the current study and are not publicly available. The data are, however, available from the authors upon reasonable request and with permission of the National Health Insurance Corporation.

## Supplementary Materials

The following are available online at https://www.mdpi.com/article/10.3390/cancers13194760/s1, Figure S1. Cumulative incidence of overall hematologic malignancy, Hodgkin’s lymphoma, and non-Hodgkin’s lymphoma in newly detected diabetes. HL, Hodgkin’s lymphoma; NHL, non-Hodgkin’s lym-phoma, Table S1. Sensitivity analysis of the association between diabetes and risk of hematologic malignancies without 1-year lag period, Table S2. The risk of hematologic malignancies according to glycemic status and diabetes duration without 1-year lag period, Table S3. Subgroup analysis of association of the risk of hematologic malignancies in diabetes patients ac-counting for diabetes medications.
