# Supplementary material for: Obesity-Independent Association between Glycemic Status and the Risk of Hematologic Malignancy: A Nationwide Population-Based Longitudinal Cohort Study
Source: Cancers (Basel). 2021 Sep 23;13(19):4760. doi: 10.3390/cancers13194760 (PMC8507554; doi:10.3390/cancers13194760)
Supplement: Supplementary file 1 [file cancers-13-04760-s001.zip › cancers-1391625-supplementary.pdf]

cancers

MDPI

Supplementary Materials: Obesity-Independent Association between Glycemic Status and the Risk of Hematologic Malignancy: A Nationwide Population-Based Longitudinal Cohort Study

Jihun Kang, Sang-Man Jin, Seok Jin Kim, Dahye Kim, Kyungdo Han, Su-Min Jeong, JiWon Chang, Sang Youl Rhee, Taewoong Choi and Dong Wook Shin

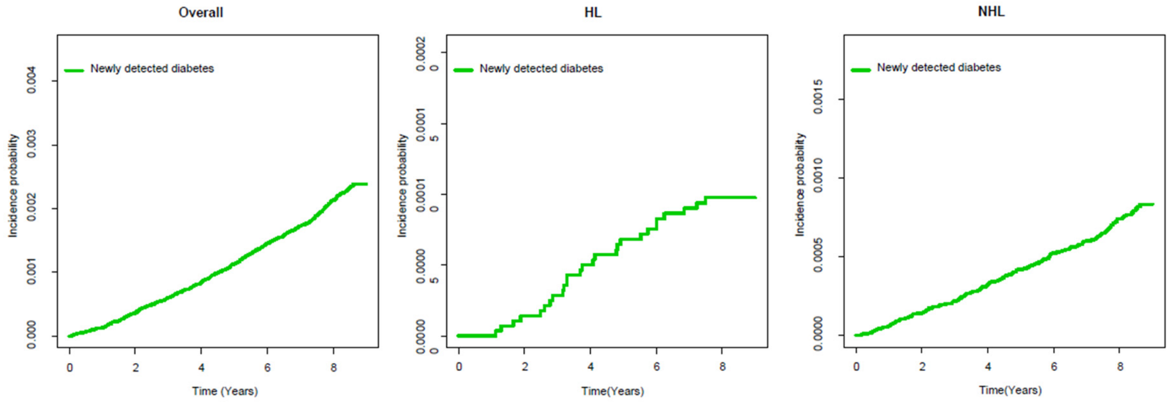

Figure S1. Cumulative incidence of overall hematologic malignancy, Hodgkin's lymphoma, and non-Hodgkin's lymphoma in newly detected diabetes. HL, Hodgkin's lymphoma; NHL, non-Hodgkin's lymphoma.

Table S1. Sensitivity analysis of the association between diabetes and risk of hematologic malignancies without 1-year lag period.

	Subjects (*N*)	Event (*n*)	Duration (person-years)	Incidence rate (per 100,000 person-years)	Model 1	Model 2
			All Hematologic Malignancy			
No Diabetes	8,945,932	13,901	70,916,233.0	19.60	1 (ref.)	1 (ref.)
Diabetes	844,161	2,312	6,248,986.4	36.99	1.12 (1.07, 1.17)	1.10 (1.05, 1.15)
			1. Hodgkin Lymphoma			
No Diabetes	8,945,932	652	68,566,621.1	1.00	1 (ref.)	1 (ref.)
Diabetes	844,161	112	6,518,828.9	1.71	1.22 (1.00, 1.49)	1.24 (1.01, 1.51)
			2. Non-Hodgkin Lymphoma			
No Diabetes	8,945,932	5,162	72,205,902.9	7.14	1 (ref.)	1 (ref.)
Diabetes	844,161	882	6,476,722.0	13.61	1.13 (1.06, 1.22)	1.11 (1.03, 1.19)
			3. Myeloid Leukemia			
No Diabetes	8,945,932	4,070	70,943,001.6	5.73	1 (ref.)	1 (ref.)
Diabetes	844,161	628	6,091,173.6	10.31	1.17 (1.08, 1.27)	1.14 (1.05, 1.24)
			4. Lymphoid Leukemia			
No Diabetes	8,945,932	1,166	71,227,855.8	16.37	1 (ref.)	1 (ref.)
Diabetes	844,161	168	6,040,775.2	27.81	1.142 (0.98, 1.34)	1.13 (0.96, 1.32)
			5. Multiple Myeloma			
No Diabetes	8,945,932	3,375	66,059,894.3	5.10	1 (ref.)	1 (ref.)

cancers

MDPI

Diabetes	844,161	603	5,815,411.3	10.36	1.06 (0.98, 1.15)	1.05 (0.97, 1.14)

Model 1was adjusted for age and sex. Model 2 was additionally adjusted for smoking, drinking, physical activity and body mass index.

Table S2. The risk of hematologic malignancies according to glycemic status and diabetes duration without 1-year lag period.

	**Subjects (*N*)**	**Event (*n*)**	**Duration (person-years)**	**Incidence rate (per 100,000 person-years)**	**Model 1**	**Model 2**
			All Hematologic Malignancy			
Normoglycemia	6,731,652	9,778	53,358,799.5	18.32	1 (ref.)	1 (ref.)
Impaired fasting glucose	2,214,280	4,123	17,539,456.3	23.50	0.99 (0.95, 1.0)	0.98 (0.95, 1.02)
Newly detected diabetes	287,175	598	2,217,196.2	26.97	1.02 (0.94, 1.10)	1.00 (0.92, 1.08)
Diabetes (<5 years)	290,305	814	2,100,862.0	38.74	1.15 (1.07, 1.23)	1.11 (1.04, 1.19)
Diabetes (≥5 years)	266,681	900	1,970,400.2	45.67	1.17 (1.10, 1.25)	1.15 (1.07, 1.23)
			1. Hodgkin Lymphoma			
Normoglycemia	6,731,652	464	51,676,133.2	0.89	1 (ref.)	1 (ref.)
Impaired fasting glucose	2,214280	187	16,804,457.2	1.11	1.00 (0.85, 1.18)	1.03 (0.87, 1.211)
Newly detected diabetes	287,175	28	2,392,957.89,	1.17	0.94 (0.64, 1.39)	0.97 (0.65, 1.43)
Diabetes (<5 years)	290,305	46	2,226,093.7	2.06	1.46 (1.08, 1.97)	1.49 (1.10, 2.01)
Diabetes (≥5 years)	266,681	39	2,032,732.2	1.91	1.24 (0.90, 1.70)	1.26 (0.91, 1.74)
			2. Non-Hodgkin Lymphoma			
Normoglycemia	6,731,652	3,565	53,998,788.3	6.66	1 (ref.)	1 (ref.)
Impaired fasting glucose	2,214,280	1,598	18,113,806.4	8.88	1.04 (0.98, 1.10)	1.02 (0.96, 1.08)
Newly detected diabetes	287,175	210	2,253,218.9	9.32	0.98 (0.85, 1.12)	0.96 (0.83, 1.10)
Diabetes (<5 years)	290,305	310	2,268,071.4	13.66	1.12 (1.00, 1.26)	1.08 (0.97, 1.22)
Diabetes (≥5 years)	266,681	361	1,994,916.0	18.09	1.29 (1.16, 1.44)	1.26 (1.13, 1.40)
			3. Myeloid Leukemia			
Normoglycemia	6,731,652	2,934	53,423,161.0	5.49	1 (ref.)	1 (ref.)
Impaired fasting glucose	2,214,280	1,135	17,501,927.5	6.48	0.95 (0.89, 1.01)	0.94 (0.87, 1.00)
Newly detected diabetes	287,175	165	2,239,413.7	7.36	0.97 (0.83, 1.14)	0.95 (0.82, 1.12)

Diabetes (<5 years)	290,305	217	1,956,188.6	11.09	1.22 (1.07, 1.38)	1.17 (1.03, 1.33)
Diabetes (≥5 years)	266,681	247	1,965,934.4	12.56	1.23 (1.08, 1.39)	1.19 (1.05, 1.35)
4. Lymphoid leukemia						
Normoglycemia	6,731,652	821	53,450,520.8	1.53	1 (ref.)	1 (ref.)
Impaired fasting glucose	2,214,280	345	17,728,674.2	1.94	1.04 (0.92, 1.18)	1.03 (0.91, 1.17)
Newly detected diabetes	287,175	50	2,136,478.2	2.34	1.15 (0.87, 1.51)	1.13 (0.86, 1.49)
Diabetes (<5 years)	290,305	59	1,865,435.7	3.16	1.29 (1.02, 1.64)	1.26 (0.99, 1.60)
Diabetes (≥5 years)	266,681	59	2,083,186.2	2.83	1.04 (0.80, 1.35)	1.02 (0.78, 1.33)
5. Multiple Myeloma						
Normoglycemia	6,731,652	2,357	50,042,462.9	4.71	1 (ref.)	1 (ref.)
Impaired fasting glucose	2,214,280	1,018	16,092,317.4	6.32	0.99 (0.92, 1.06)	0.99 (0.92, 1.06)
Newly detected diabetes	287,175	158	2,014,021.7	7.84	1.07 (0.92, 1.25)	1.07 (0.93, 1.25)
Diabetes (<5 years)	290,305	219	1,997,081.9	10.96	1.09 (0.96, 1.24)	1.07 (0.94, 1.21)
Diabetes (≥5 years)	266,681	226	1,825,230.2	12.38	1.02 (0.90, 1.161)	1.01 (0.89, 1.14)

Model 1 was adjusted for age and sex. Model 2 was additionally adjusted for, smoking, drinking, physical activity and body mass index.

Table S3. Subgroup analysis of association of the risk of hematologic malignancies in diabetes patients accounting for diabetes medications.

	Subjects (*N*)	Event (*n*)	Duration (person-years)	Incidence rate (per 100,000 person-years)	Model 1	Model 2	Model 3
All Hematologic Malignancy							
Diabetes (<5 years)	289,846	740	2,067,385.4	35.79	1 (ref.)	1 (ref.)	1 (ref.)
Diabetes (≥5 years)	266,260	818	1,860,258.4	43.97	1.09 (0.99, 1.20)	1.10 (1.00, 1.21)	1.07 (0.97, 1.19)
1. Hodgkin Lymphoma							
Diabetes (<5 years)	289,846	42	2,068,831.9	2.03	1 (ref.)	1 (ref.)	1 (ref.)
Diabetes (≥5 years)	266,260	35	1,861,752.6	1.87	0.82 (0.53, 1.27)	0.80 (0.52, 1.24)	0.79 (0.50, 1.25)
2. Non-Hodgkin Lymphoma							
Diabetes (<5 years)	289,846	282	2,068,219.3	13.63	1 (ref.)	1 (ref.)	1 (ref.)
Diabetes (≥5 years)	266,260	328	1,861,162.3	17.62	1.18 (1.01, 1.38)	1.18 (1.01, 1.38)	1.20 (1.02, 1.42)

cancers MDPI

			3. Myeloid Leukemia				
Diabetes (<5 years)	289,846	197	2,068,637.3	9.52	1 (ref.)	1 (ref.)	1 (ref.)
Diabetes (≥5 years)	266,260	224	1,861,461.8	12.03	1.13 (0.93, 1.36)	1.15 (0.95, 1.39)	1.05 (0.86, 1.28)
			4. Lymphoid leukemia				
Diabetes (<5 years)	289,846	54	2,068,839.1	2.61	1 (ref.)	1 (ref.)	1 (ref.)
Diabetes (≥5 years)	266,260	54	1,861,744.1	2.90	0.88 (0.62, 1.26)	0.89 (0.62, 1.28)	0.96 (0.66, 1.40)
			5. Multiple Myeloma				
Diabetes (<5 years)	289,846	199	2,068,565.9	9.62	1 (ref.)	1 (ref.)	1 (ref.)
Diabetes (≥5 years)	266,260	205	1,861,468.3	11.01	1.01 (0.85, 1.21)	1.01 (0.85, 1.22)	1.00 (0.82, 1.20)

Model 1 was adjusted for age and sex. Model 2 was additionally adjusted for smoking, drinking, physical activity and body mass index. Model 3 was additionally adjusted for diabetes medications.
